# Increased Foraging in Outdoor Organic Pig Production—Modeling Environmental Consequences

**DOI:** 10.3390/foods4040622

**Published:** 2015-11-02

**Authors:** Malene Jakobsen, Teodora Preda, Anne Grete Kongsted, John Erik Hermansen

**Affiliations:** Department of Agroecology, Faculty of Science and Technology, Blichers Allé 20, Aarhus University, P.O. Box 50, Tjele DK-8830, Denmark; E-Mails: teodora.preda@agro.au.dk (T.P.); anneg.kongsted@agro.au.dk (A.G.K.); john.hermansen@agro.au.dk (J.E.H.)

**Keywords:** organic, pig production, foraging, nitrogen balance, greenhouse gas emission, carbon footprint, soil carbon emission, Indirect Land Use Change

## Abstract

Consumers’ motivations for buying organic products include a wish of acquiring healthy, environmentally friendly products from production systems that also ensure a high level of animal welfare. However, the current Danish organic pig production faces important challenges regarding environmental impact of the system. High ammonia emissions arise from outdoor concrete areas with growing-finishing pigs and sows on pasture possess an increased risk of nitrogen (N) leaching. Direct foraging in the range area is suggested as a way to improve the nutrient efficiency at farm level and to support a more natural behavior of the pig. Thus, by modeling, we investigated the environmental consequences of two alternative scenarios with growing-finishing pigs foraging in the range area and different levels of crops available for foraging; grass-clover (lowest integration of forage) or a combination of lucerne, grass-clover and Jerusalem artichokes (highest integration of forage). It was possible to have growing-finishing pigs on free-range without increasing N leaching compared to the current practice. The alternative system with lucerne, grass-clover and Jerusalem artichokes showed the lowest carbon footprint with 3.12 CO_2_ eq kg^−1^ live weight pig compared to the current Danish pasture based system with 3.69 kg CO_2_ eq kg^−1^ live weight pig. Due to positive impact on soil carbon sequestration, the second alternative system based on grass-clover  showed a similar carbon foot print compared to current practice with 3.68 kg CO_2_ eq kg^−1^ live weight pig. It is concluded that in practice there is room for development of organic pig production systems where direct foraging plays a central role.

## 1. Introduction

In general, society and consumers are positive towards organic farming. The European Union supports organic farming as a sustainable form of agricultural production, providing public goods such as animal welfare, environmental protection, increased biodiversity and contributing to rural development [1]. For consumers, motivations for buying organic have been found to be related to a value-based wish of acquiring healthy, environmentally friendly products from production systems that also ensure a high level of animal welfare [2]. Regarding willingness to pay for organic pork products, a recent investigation reported that production methods in terms of organic or conventional did not have any effect but premium production did [3]. In this context premium pork production was described by words such as free-range, locally produced, foraging in the field and high quality feed. These qualities are described as intrinsic as opposed to extrinsic qualities (visual appearance of the meat) the latter only being marginally significant for organic pork. Hence, in terms of increasing and diversifying the alternative pig production, which in Denmark at the moment is marginal, the above findings advocate for development of an organic pig production that includes such intrinsic qualities.

Organic agriculture is based on the principles of Health, Ecology, Fairness and Care. Among others, these basic principles focus on the production of nutritious high quality food that contributes to preventative health care and well-being for animals and humans. In addition, recirculation of nutrients in the farming system and use of local renewable resources is a central part of the principles [4]. Furthermore, emphasis is on providing animals with opportunities to perform natural behavior, getting feed adapted to their physiology and live in a natural environment [5]. These principles are reflected in the European organic regulations, e.g., “*…feeding of livestock with organic-farming crop products produced on the holding itself or on neighbouring organic holdings*” and “...*animals should have*, *whenever possible*, *access to open air or grazing areas*” [1]. In the current Danish organic pig production, sows are on pasture all year round and in general animals have more space compared to conventional production. Furthermore, pigs have access to roughage and rooting material such as straw. These requirements are thought to be some of the reasons for the low antimicrobial use in organic pig production [6,7].

On the other hand, important challenges are related to the environmental impact of organic production systems. Typically, in Denmark, growing pigs are housed indoors with access to outdoor concrete yards [8] and the consequence is high ammonia emissions from the outdoor area [9]. The practice of keeping sows on pasture all year round constitutes a significant risk in terms of nitrate leaching and ammonia emissions [10]. Nitrogen (N) surpluses per hectare have been found to range between 264 and 500 kg N ha^−1^ [11,12]. The typical organic pig production system has been estimated to cause 21%–65% more eutrophication and acidification kg^−1^ pig compared to conventional indoor production [13], whereas impact on global warming is comparable with that of conventional production [14]. In terms of animal welfare, the Danish organic pig production system also possesses relevant concerns related to the ability of pigs to perform species-specific behavior. Indoor housing of growing—finishing pigs and snout-ringing of sows to prevent them from destroying the sward clearly conflicts with consumers’ expectations of livestock being outdoors and able to perform natural behavior [15].

Despite the obvious intrinsic qualities related to outdoor production, organic growing-finishing pigs on pasture are rare in Denmark. This is partly due to the well-documented negative environmental side effects of this type of production [16,17] combined with high feed costs as a consequence of an increased input of purchased supplementary feed. Halberg *et al.* (2010) [13] concluded that one obvious way to improve the nutrient efficiency of free-range pig production is to increase nutrient intake from direct foraging in the range area. Thereby, the need for large input of nitrogen into the free-range system via supplemental feed is reduced. This increases nutrient recirculation in the farming system and thus, decreases risk of nitrogen leaching. Concurrently, it may even pose new market opportunities for organic pork with clear intrinsic qualities justifying premium prices.

The pig is an opportunistic omnivorous animal with an inherent motivation for foraging as described by Andresen (2000) and Beattie and Connell (2002) [18,19]. Hence, it seems obvious to try and motivate free-range pigs to increase food intake from the areas they occupy. From studies on direct foraging in pregnant sows, uptake of around 40%–65% of energy requirements from clover–grass has been documented [20,21,22]. Regarding direct foraging in growing pigs, studies have reported an intake of up to 20% of energy requirements by grazing [23,24,25,26,27]. In addition, intake of Jerusalem artichokes have been estimated to amount to 60% of energy requirements [28] and in a recent study, growing pigs were estimated to have a daily lucerne dry matter intake corresponding to 47% of total lysine intake (supplementary feed and lucerne) [29]. Furthermore, Kongsted *et al.* (2015) [30] found that pigs were able to obtain vitamins and minerals by direct foraging in the range area. In this context, it is of major relevance that Jerusalem artichokes [28] as well as lucerne [31] are high yielding crops. These studies indicate that direct foraging can pose an important contribution to pigs’ energy and nutrient requirements. However, outdoor rearing is related to increased energy requirements, corresponding to an increase in annual feed requirements of approximately 15% under Northern European conditions [32]. Likewise, making appropriate areas available for foraging has an impact on total land use and crop rotation. Thus, the overall technical and environmental impact of establishing organic pig production systems where a larger proportion of the feed intake is achieved by direct foraging remains unknown.

Against this background, the present paper investigated—by modeling—the technical and environmental performance at farm level of rearing free-range growing-finishing pigs foraging directly in the range area as compared to the current Danish organic pig production system with sows on pasture all year round and growing-finishing pigs housed indoors. Two alternative scenarios were assessed. Alternative 1 (*Free-range: grass-clover*) represents a system where both sows and growing-finishing pigs are foraging on grass-clover pastures all year round, whereas alternative 2 (*Free-range: alternative crops*) is a scenario representing direct foraging on lucerne, grass-clover and Jerusalem artichokes.

## 2. Materials and Methods

### 2.1. Three Modeled Scenarios for Organic Pig Production

Three scenarios for Danish organic pig production systems were modeled based on a synthesis of key figures from organic pig farms, empirical data from on farm studies and experimental data, organic as well as conventional.

The three types of organic pig production systems considered were; the reference scenario(*Indoor finishing*), alternative scenario 1 (*Free-range: grass-clover*) and alternative scenario 2 (*Free-range: alternative crops*). According to Danish environmental regulation, the general area requirement is 1.4 livestock units (LSU) per hectare (ha) [33]. One LSU equals the production of 100 kg N in animal manure. However, for organic pig production, a stocking rate of 2.8 LSU ha^−1^ (280 kg N ha^−1^), every *second year*, is allowed if a nitrogen demanding crop is sown in between years with pig production [33]. On this basis, the starting point for all three scenarios was 84 ha and 100 annual sows with production of 1925 finishers (Table 1).

**Table 1:** Production characteristics for three organic pig production systems.

Production Characteristics:	Sow Herd	Growing Pigs		
	All Systems	Indoor Finishing ^1^	Free-Range: Grass–clover ^2^	Free-Range: Alternative Crops ^3^
Annual sows	100			
Growing-finishing pigs produced 110 kg		1925	1925	1925
Crop rotation, ha				
Barley	12	32	24	22
Oats	12			
Peas		16		
Grass–clover	12		24	6
Lucerne				10
Jerusalem artichokes				10
Total hectares	36	48	48	48
Yield, kg DM ha^−1^				
Barley	3825	3825	3825	3825
Oats	3825			
Peas		2,556		
Grass–clover (thereof grazed)	4920 (1,630)		4094 (1356)	2326 (2,326)
Lucerne (thereof grazed)				6531 (1,454)
Jerusalem artichokes				6667
Average yield, kg DM ha^−1^	4190	3402	3960	4793

^1^: Indoor finishing: Sows on pasture and growing-finishing pigs housed indoors; ^2^: Free-range: grass-clover: Sows on pasture and growing-finishing pigs foraging on grass-clover fields; ^3^: Free-range: alternative crops: Sows on pasture and growing-finishing pigs foraging on lucerne, grass-clover and Jerusalem artichokes.

**Figure 1:**
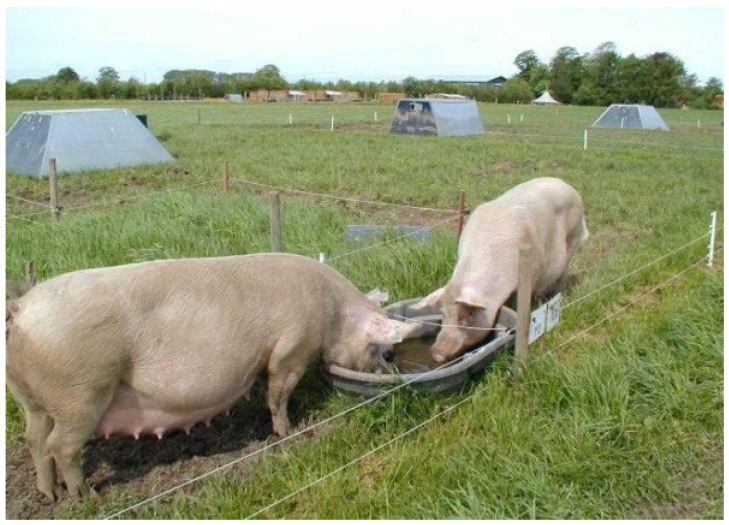
Lactating free-range sows.

**Figure 2:**
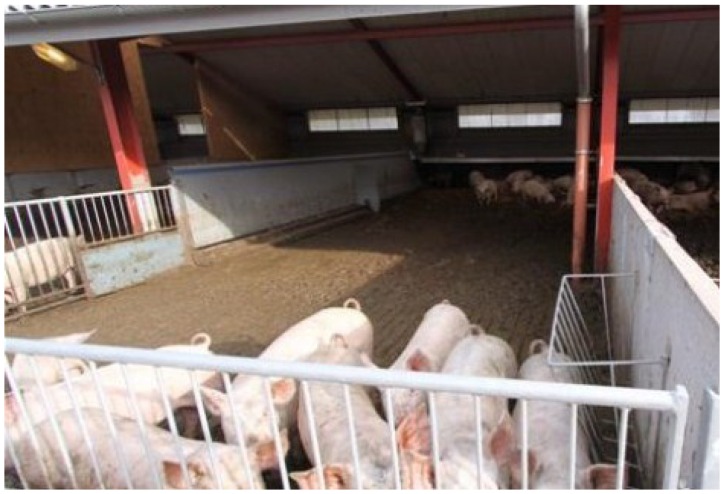
Growing-finishing pigs in outdoor concrete area.

In the *Indoor finishing* scenario (Figure 1), pregnant and lactating sows are free-range on grassland with access to insulated huts for protection, which are placed directly on the ground and supplied with straw. Sows are moved in the crop rotation between years and every third year they return to the same field. Sows for insemination are housed indoors in a loose house system for approximately five days during each production cycle. Growing-finishing pigs are housed indoors with access to outdoor concrete areas until slaughter at 110 kg live weight (Figure 2). Weaned pigs are housed in smaller groups (30 weaners) until approximately 30 kg live weight and subsequently moved to larger groups (50 growing-finishing pigs). The indoor housing has natural ventilation and a lying area supplied with straw and a cover. Part of the indoor and outdoor concrete floor is slatted, which allows for slurry to be collected. The system is relatively well described in terms of housing design and management but also regarding production results [34].

**Figure 3:**
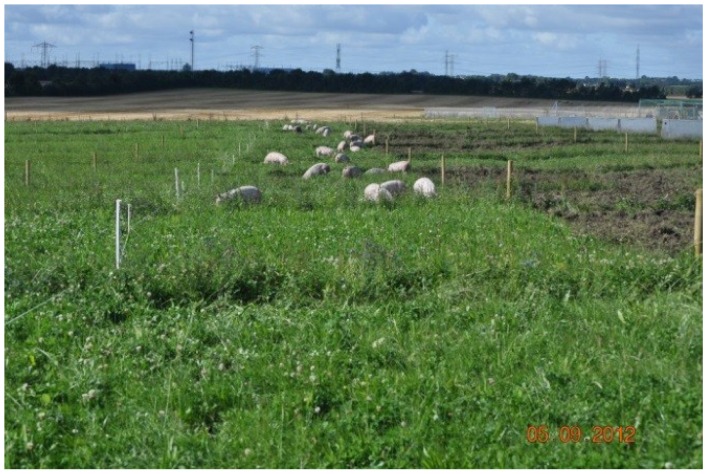
Growing-finishing pigs foraging on grass-clover.

**Figure 4:**
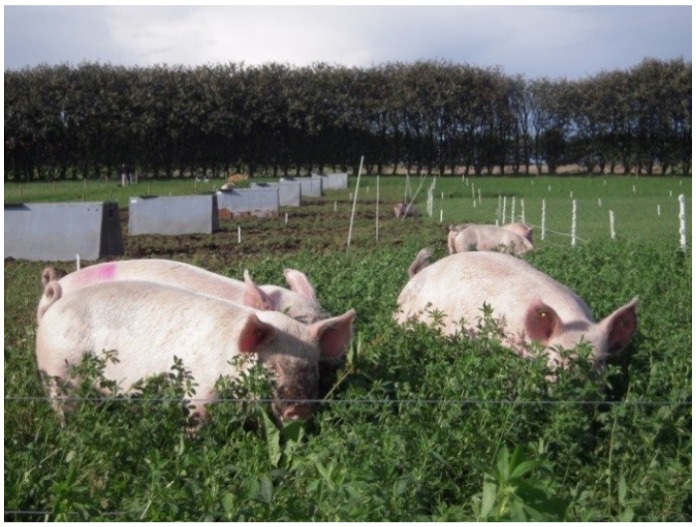
Growing-finishing pigs foraging on lucerne.

**Figure 5:**
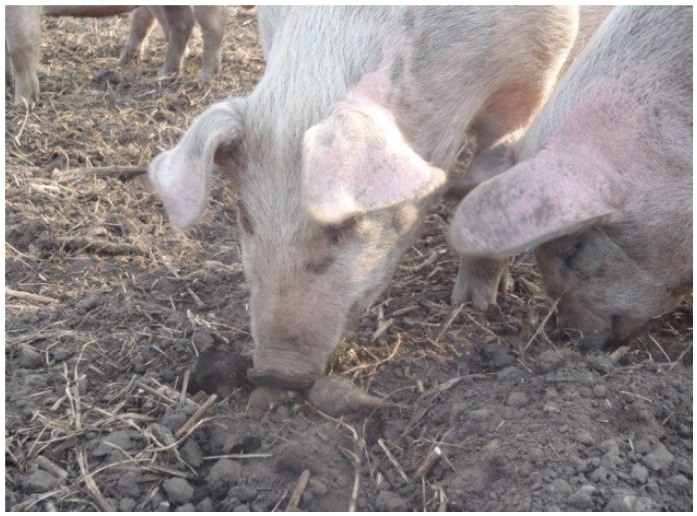
Growing-finishing pigs foraging on Jerusalem artichokes.

In the *Free-range: grass-clover* scenario (Figure 3), growing-finishing pigs are reared on grass–clover fields all year round and thus, no collection of manure is taking place. Hence, facilities for indoor housing are reduced and instead pigs have access to insulated huts, which are placed directly on the pasture and supplied with straw. Pigs are moved in the crop rotation between years. In the second alternative scenario (*Free-range: alternative crops*) (Figure 4 and Figure 5), growing-finishing pigs are reared on fields with lucerne, grass–clover and Jerusalem artichokes. The housing is similar to the *Free-range: grass-clover* scenario. Pigs are moved in the crop rotation between years. Also, pigs are moved throughout the year depending on availability of the various crops. In the alternative scenarios, direct foraging in the areas the pigs occupy is a key concept. Growing-finishing pigs are subjected to strip-grazing, which prevents the pigs from quickly deteriorating the sward. The sow herd system is similar to the *Indoor finishing* scenario.

### 2.2. Key-Figures and Technical Results

#### 2.2.1. Animal Production

Selected key figures for the sow herd and growing-finishing pigs in all three scenarios are presented in Table 2. The number of farrowings per sow in organic production is 1.9 litter sow^−1^ year^−1^ [35], which is lower compared to conventional production due to a longer lactating period in the organic system. Percentage of first parity sows was assumed to be 23.5%, which is similar to conventional production [36] since organic pig producers use similar breeding material. Gilts are recruited from own herd in relation to fattening pigs being selected for slaughter at 110 kg live weight. Gilts are inseminated at 7½–9 months of age corresponding to a weight of approximately 130–140 kg. In total, mortality rate for weaners (13.8–30 kg), growers (30–50 kg) and finishers (50–110 kg) is 6.9% [35]. Taking into account weaned piglets per annual sow, mortality figures and replacement gilts, 1925 finishers are produced per year for slaughter.

**Table 2:** Key figures for the sow herd and growing-finishing pigs in three organic pig production systems.

**Sow Herd**	**Key Figures**
Farrowings, no of litters/sow	1.9
Lactation period, days	51
Live born piglets, no per farrowing	13.7
Piglet mortality, in percentage of total number of live born piglets	20.9
Weaned piglets/annual sow, no	21.1
Piglet live weight at weaning, kg	13.8
Sow mortality, in percentage of total number of annual sows	5
First parity sows, in percentage of total number of annual sows	23.5
Voluntarily culled sows, in percentage of total number of annual sows	44.7
Weight at insemination, kg	130
**Growing-finishing pigs**	**Key Figures**
Mean daily weight gain, g	811
Mortality weaners, in percentage of total number of weaners	4
Mortality growing pigs, in percentage of total number of growing pigs	1.9
Mortality finishers, in percentage of total number of finishers	0.9
Live weight at slaughter, kg	110

#### 2.2.2. Feed Consumption

According to European organic regulations, it is prohibited to add synthetic amino acids to the feed [1]. Hence, in order to fulfill the essential amino acid requirements of organic pigs, farmers and feed mills are forced to optimize feed mixtures by use of non-synthetic protein feedstuffs. Consequently, organic feed mixtures usually contain relatively high amounts of protein compared to the pigs’ biological needs. Therefore, rather than calculating *nutrient requirements*, the point of departure was the *nutrient content* in organic concentrate feed mixtures purchased at Danish feed mills [37], as this was assessed to be a more correct indication of the actual amount of N deposited in the systems. Furthermore, as self-sufficiency is of high priority in organic farming, protein sources were, as much as possible, based on crops cultivated in Denmark or Northern Europe. Since weaners and lactating sows are the most demanding groups of animals in terms of essential amino acid requirements, it was decided to import concentrate feed mixtures from outside the farm to these groups. The feed produced on farm was distributed between the remaining groups of pigs according to their nutrient consumptions. The remaining feed was imported from outside the farm. Feed compositions were identified based on the recommended level of digestible crude protein per megajoule metabolizable energy (MJ^−1^ ME) according to Danish norms for conventional pigs [38].

In Table 3 the energy and protein consumption for the various groups of animals in the three scenarios is presented. For sows, the point of departure was conventional energy norms in free-range production for maintenance, fetal growth and milk production [39,40]. On top of this was added 15% related to energy use for thermoregulation and a higher activity in organic production compared to conventional as described by Edwards (2003) [32]. In addition, for growing-finishing pigs, energy and protein consumption was based on conventional norms [41,42]. In the *Indoor finishing* scenario, 7% was added on top of the conventional energy norm in order to meet energy requirements related to thermoregulation and increased activity. During cold periods natural ventilation in indoor housing decreases the temperature compared to conventional indoor housing. In addition, organic growing-finishing pigs have more than twice the space to roam compared to conventional pigs. In the *Free-range: grass–clover* and *Free-range: alternative crops* scenarios, energy requirements for growing-finishing pigs were increased compared to the *Indoor finishing* scenario. On top of the conventional norms, energy corresponding to an extra 20% was added due to an increased activity related to direct foraging of various crops and thermoregulation (Table 3).

In organic production all pigs must have access to roughage either by direct foraging or by allocation of roughage [43]. In the *Indoor finishing* system, weaners, growers and finishers were allocated grass–clover silage corresponding to 3% of the total daily energy in the feed ration, which is the reported level of intake when pigs are not restricted in concentrate feed [44]. As opposed to growing and finishing pigs, it was assumed that weaners were not able to utilize energy and nutrients contained in the silage since the digestive system at this point has not developed the capacity to digest fiber-rich feed. Pregnant, dry sows and gilts were allocated the remaining production of silage. Together with the production of grass–clover in the paddocks this comprised 22% of the total amount of energy in the feed ration for non-lactating sows. In the *Free-range: grass-clover* scenario a large amount of silage was produced in order to allow sufficient area for free-range foraging and thus 36% of the total energy in the feed ration for pregnant, dry sows and gilts comprised of grass-clover. In the *Free-range: alternative crops* scenario, overall 60% of the total energy in the feed ration of pregnant sows consisted of grass–clover and Jerusalem artichokes (see supporting material). The maximum allocation of forage to growing pigs and finishers is generally not well documented and we have based our assumptions on recent literature. Bikker and Binnendijk (2012) [45] reported an intake of grass silage by growing pigs and finishers corresponding to 6% and 15%, respectively, on a DM basis, increasing to 10% and 20% of DM in the late period of the growing and finishing stage, respectively. This resulted in a slight reduction (4%) in daily gain compared to a diet without silage. Furthermore, Weltin *et al.* (2014) [31] found an intake of lucerne silage corresponding to 20% and 40% for growing pigs (initial and middle period, respectively) and 50% for finishers on a dry matter basis. In this work the composition of concentrate feed was adapted to the expected intake of lucerne silage and planned to support a growth rate of 700 g day^−1^ at the beginning and end of the feeding period and 750 g in the middle period. Thus, the situation was not *ad libitum* concentrate feeding. Daily gain was significantly reduced only in the middle period and overall, the fattening period was extended with 7 days (or 5%). Under grazing conditions, Jakobsen *et al*. (2015) [29] found that for finishers, intake of lucerne and grass-clover amounted to 14.6 and 11 MJ ME kg^−1^ weight gain, respectively, when concentrate feeding (and daily gain) was reduced. On this basis, in the present study, it was estimated that in both alternative systems, *growing pigs* were able to utilize forage crops corresponding to 3.7 MJ ME kg^−1^ weight gain (up to 18% on a DM basis). For *finishers*, intake of forage was estimated to 8.5 MJ ME kg^−1^ weight gain (up to 22% on a DM basis; see supporting material) while assuming no significant impact on daily gain. Regarding forage nutrient quality, the figures used were based on Danish growing conditions [46,47]. 

**Table 3:** Total energy (maintenance, growth, fetus, lactation, activity, and thermoregulation) and crude protein (CP) consumption for sows and growing-finishing pigs in three organic pig production systems. Figures in brackets designate energy consumption related to thermoregulation and activity and they have been added to the figures already given.

	Indoor Finishing ^1^	Free-Range: Grass–clover ^2^	Free-Range: Alternative Crops ^3^
Energy consumption sow^−1^ day^−1^, megajoule metabolizable energy (MJ ME):			
Gestating	37.4	37.4	37.4
Lactating	142.1	142.1	142.1
Dry	37.4	37.4	37.4
Gilts ^4^	37.4	37.4	37.4
Energy consumption annual sow^−1^, MJ ME	23,684	23,684	23,684
Energy consumption growing-finishing pigs, MJ ME kg^−1^ weight gain:			
Weaners 13.8–30 kg	26.1 (1.7)	29.3 (4.9)	29.3 (4.9)
Growers 30–50 kg	28 (1.8)	31.7 (5.49)	31.7 (5.49)
Finishers 50–110 kg	40.2 (2.4)	45.1 (7.3)	45.1 (7.3)
Energy consumption pig^−1^, MJ ME	3532	3966	3966
Total energy consumption annual sow^−1^, MJ ME ^5^	91,852	100,228	100,228
Total CP consumption annual sow^−1^, kg CP ^6^	1231	1231	1231

^1^: Indoor finishing: Sow herd on pasture and growing pigs housed indoors; ^2^: Free-range: grass-clover: Sow herd on pasture and growing pigs foraging on grass-clover; ^3^: Free-range alternative crops: Sow herd on pasture and growing pigs foraging on lucerne, grass-clover and Jerusalem artichokes; ^4^: Energy consumption from 110 to 130 kg; ^5,6^: Including 19.3 growing-finishing pigs produced per annual sow.

#### 2.2.3. Crop Rotations and Crop Production

The range area for outdoor production of pigs was calculated according to Danish environmental regulation [33]. Thus, for the sow herd in each scenario, a total minimum area for pastures of 10 ha was required for pregnant and lactating sows, and the area must not be grazed or given any kind of manure the following year. In order to optimize the crop rotation related to the sow herd, sows returned every third year to the same field instead of every second year. In all three scenarios the crop rotation for the sow herd and growing-finishing pigs amounted to 36 and 48 ha, respectively, as presented in Table 1.

In the *Indoor finishing* scenario, the crop rotation related to growing-finishing pigs consisted of 16 ha of spring barley, followed by 16 ha of peas and afterwards 16 ha of barley (Table 1). The total production of oats was allocated to pregnant, dry sows and gilts. Growers and finishers received the entire production of barley and peas.

Growing-finishing pigs were foraging directly in the areas they occupied in the *Free-range: grass-clover* and *Free-range: alternative crops* scenarios. In the *Free-range: grass-clover* scenario half of the crop rotation was cultivated with barley and the other half with grass-clover. The growing-finishing pigs were foraging on the 24 ha with grass-clover, which were divided into four paddocks each consisting of 6 ha. Grass–clover fields were either grazed and or cut for silage production. Since farrowings were evenly spread across the year not all growing-finishing pigs had access to fresh pasture, so during winter they were supplied with grass-clover silage.

In the *Free-range: alternative crops* scenario the 48 ha were divided into two crop rotations. The first rotation consisted of 2 × 5 ha with lucerne, followed by 2 × 5 ha with barley and afterwards 2 × 5 ha of Jerusalem artichokes. The second rotation comprised 18 ha with 6 ha of barley, followed by 6 ha of grass–clover and then 6 ha of barley. During January, February and December it was assumed that growing-finishing pigs were supplemented with lucerne silage. In March, April, September, October and November, they were assumed to forage on Jerusalem artichokes. During summer months, they were foraging on grass-clover (May and June) and lucerne (July and August). In the *Free-range: grass-clover* scenario as well as the *Free-range: alternative crops* scenario, the total production of barley and oats were allocated to the growing-finishing pigs. Fields with Jerusalem artichokes are productive within the year of planting seed tubers. In addition, tubers can endure frost, overwinter and be harvested by the pigs the following year. Lucerne is under-sown with barley as a cover crop and pigs are foraging within the first year of sowing. However, due to pigs’ rooting activities it was assumed that no regrowth is taking place. Thus, pigs cannot return to the same area the following year.

Estimated average crop production in all three scenarios is presented in Table 1. For the sow herd, pastures for lactating sows (2.5 ha) were assumed to not contribute in terms of grazing or silage production. 2.5 ha of pastures for pregnant sows were estimated to produce 18,300 MJ ME ha^−1^. In addition, 5 ha grazed after silage production was assumed to produce 18,300 MJ ME ha^−1^. The 5 ha with silage produced 30,500 MJ ME ha^−1^. Estimated average yields of cereals and peas were based on figures reported from organic dairy fields with loamy sand and sparse irrigation [48]. Straw yield was 1600 and 2000 kg ha^−1^ for barley and oats, respectively [49].

In all three scenarios, straw use was 220, 7.5 and 40 kg per annual sow, weaner and growing pig, respectively, according to estimated use in organic production systems [50]. The remaining straw (wheat) requirements were imported from outside the farm.

### 2.3. Nitrogen Balance

The nitrogen balance was estimated at herd, field and farm level as the difference between the input (imported feed, imported straw, N biological fixation, deposition) and output (live pigs) as described by Nielsen and Kristensen (2013) [51].

The biological N fixation was assumed to be 150 kg N ha^−1^ for grass-clover silage and 30 kg N ha^−1^ for grass-clover grazed. The estimated biological N fixation for peas and lucerne was 41 and 42 kg N tons^−1^ dry matter harvested, respectively [52]. Nitrogen deposition for Denmark is 16 kg N ha^−1^ [13].

Nitrogen leaching was estimated by deducting N losses and soil N changes from N surplus. The calculated N losses were ammonia volatilization and denitrification, which were estimated as given in Table 4. Soil N changes were calculated according to the method described by Petersen *et al.* (2013) [53] and Mogensen *et al.* (2014) [54] by considering net crop yield, crop residues and losses in the field. Crop losses in grazing areas with pigs were estimated to be 60%, which is higher than crop losses in fields with grazing cattle (40%) due to a different grazing behavior (including rooting) by pigs.

**Table 4:** Factors for estimation of emissions from pig and crop production in three organic pig production systems.

Type of Emissions	Source of Emissions	Amount	Emission Factor	Reference
NH_3_-N, kg	Stable-slurry	kg N in manure ex animal	0.26	[9]
	Storage-slurry	kg N in manure ex stable	0.027	[55]
	Application-slurry	kg N in manure ex storage	0.12	[55]
	Grazing	kg N in feed input	0.13	[10]
	Crop residues-grass		0.5 ^1)^	[56]
	Crop residues-other crops		2 ^1)^	[56]
N_2_O-N direct, kg	Stable	kg N in manure ex animal	0.002	[57]
	Storage	kg N in manure ex stable	0.005	[57]
	Application-slurry	kg N in manure ex storage	0.01	[57]
	Grazing	kg N in manure deposited at pasture	0.02	[57]
	Crop residues	kg N in crop residues per ha	0.01	[57]
N_2_O-N indirect, kg	Ammonia	kg NH_3_-N	0.01	[57]
	N-leaching	kg NO_3_-N	0.0075	[57]
CH_4_, kg	Storage-in house storage	kg volatile solids in slurry	0.03	[57]
	Storage-outside storage with natural crust	kg volatile solids in slurry	0.1	[57]
	Grazing	kg manure deposited at pasture	0.01	[57]
	Enteric fermentation:			
	Sows	kg feed	0.002 ^2)^	[58]
	Growing pigs	kg feed	0.001 ^3)^	[58]

^1)^: kg N ha^−1^ year^−1^; ^2)^: 0.002 = 100 g ResDkg feed × 1340 J g^−1^ ResD/(55.65 MJ kg^−1^ CH4 × 10^6^ J MJ^−1^); ^3)^: 0.001 = 90 g ResD/kg feed x 670 J g^−1^ ResD/(55.65 MJ/kg CH4 × 10^6^ J MJ^−1^; ResD: digested fiber ingested.

### 2.4. Greenhouse Gas Emissions

The estimation of greenhouse gas (GHG) emissions from the three production systems included three main categories of GHG: nitrous oxide (N_2_O) (from feed production), methane (CH_4_) (from enteric fermentation and from manure management) and carbon dioxide (CO_2_) (from feed production, soil C changes and land use change (LUC)).

The contribution of enteric fermentation to methane emissions was calculated according to the methodology described by Rigolet *et al.* (2010) [58], as given in Table 4. Digested fiber ingested (ResD) for sows and piglets was assumed to be 100 and 90 g kg^−1^ feed, respectively [58].

Methane emissions from manure management were deduced and based on IPCC (2006) [57] methodology. Accordingly, methane emissions depend on the content of volatile solids in manure. The content of volatile solids for the three systems was calculated according to Nguyen *et al.* (2011) [59]. The emission factors vary in relation to manure management (3% for slurry in-house storage less than one month, 10% for slurry outside storage with natural crust cover, 1% for grazed areas).

The emissions at farm level (CO_2_ from on-farm operations, C sequestration and land use change) related to the organic home-grown feeds were estimated according to Mogensen *et al.* (2014) [54]. The consumption of inputs ha^−1^ (diesel, lubricant, electricity) during on-farm operations was assumed to be similar for grass–clover and lucerne. For Jerusalem artichokes, it was assumed that consumption of inputs was similar to consumption of fodder beet. In order to estimate impacts of land use change the methodology suggested by Audsley *et al.* (2009) [60] was implemented. Thus, each type of crop production (whether home-grown or imported) was assumed to increase pressure on land use according to the area occupied by the factor 143 g CO_2_ m^−2^.

Figures for carbon footprint (CF), soil C changes and LUC of the organic imported feed were deduced from Mogensen *et al.* (2014) [54]. It was assumed that oats and wheat had similar impacts as barley and faba beans to peas. In relation to CF of milk powder, the figure from Flysjö (2012) [61] was used (8.2 kg CO_2_ eq kg^−1^ skimmed milk powder).

## 3. Results and Discussion

### 3.1. Imported Supplementary Feed

Comparing the three scenarios in terms of the need for imported MJ ME in supplementary feed per annual sow, the highest level was found in the *Free-range: grass–clover* scenario with 55,299 MJ ME annual sow^−1^, which was 6.6% higher compared to the *Indoor finishing* scenario (Table 5). Thus, despite higher overall crop yields in the free-range scenarios (Table 1), this was more than counterbalanced by the increased energy requirements for activity and thermoregulation compared to the *Indoor finishing* scenario. The reduced import of MJ ME in the *Free-range: alternative crops* scenario compared to the *Free-range: grass–clover* scenario was on the other hand related to the production of the high-energy containing forage crop Jerusalem artichokes in the former scenario. 

In terms of total kg CP imported per annual sow, clearly the highest level was found in the *Indoor finishing* scenario with 865 kg imported CP per annual sow. In comparison, 734 and 749 kg N was imported per annual sow in the *Free-range: grass–clover* and the *Free-range: alternative crops* scenarios, respectively. The reduced import of CP in the alternative scenarios was due to a larger area being grown with protein-rich crops such as grass–clover and lucerne as a result of a higher estimated consumption of forage by the sow herd as well as growing pigs compared to the *Indoor finishing* scenario.

As a consequence of the reduced area with cereals in the alternative systems, less straw was available and thus more straw was imported (372 and 404 kg per annual sow in the *Free-range: grass–clover* and the *Free-range: alternative crops* scenario, respectively) compared to 244 kg per annual sow in the *Indoor finishing* scenario.

**Table 5:** Imported feed in MJ metabolizable energy (ME) and kg crude protein (CP) (brackets) per annual sow in three organic pig production systems.

	Indoor Finishing ^1^	Free-Range: Grass–clover ^2^	Free-Range: Alternative Crops ^3^
Feed item			
Barley	12,449 (79)	21,371 (136)	22,279(142)
Oats	3055 (24)	6899 (54)	3123 (24)
Wheat	3069 (20)	3069 (20)	3069 (20)
Rapeseed oil	976 (0)	1,074 (0)	1084 (0)
Rapeseed cake	10,281 (286)	7695 (213)	2311 (63)
Peas	9280(150)	6513 (105)	1911 (31)
Faba beans	10,913 (252)	6693 (155)	18,111 (418)
Soy cake	760 (24)	760 (24)	760 (24)
Skimmed milk powder	1113 (30)	1225 (27)	1236 (27)
Total amount of MJ ME and (CP) in imported feed per annual sow	51,896 (865)	55,299 (734)	53,884 (749)
Imported energy and CP in percentage of energy and CP consumption per annual sow	56.4 (70)	55.1 (59.6)	53.7 (60.8)

^1^: Indoor finishing: Sows on pasture and growing-finishing pigs housed indoors; ^2^: Free-range: grass-clover: Sows on pasture and growing-finishing pigs foraging on grass-clover; ^3^: Free-range: alternative crops: Sows on pasture and growing-finishing pigs foraging on lucerne, grass-clover and Jerusalem artichokes.

### 3.2. Nitrogen Leaching at Farm Level

Nitrogen balances (kg N ha^−1^) for the three systems are presented in Table 6. Overall, in the alternative scenarios the level of imported feed N was reduced (145 and 140 kg N ha^−1^ for the *Free-range: grass-clover* and the *Free-range: alternative crops* scenarios, respectively), compared to the *Indoor fattening* scenario with 164 kg N ha^−1^ corresponding to a 15% decrease. The reduction in N input from imported supplementary feed was to some degree counteracted by higher figures for biological fixation in the alternative systems. Nitrogen input from biological fixation was 25% lower when all categories of animals grazed on grass–clover in the *Free-range: grass-clover* scenario compared to the *Free-range: alternative crops* scenario where sows and piglets grazed grass-clover pastures and growing pigs were foraging on grass-clover, lucerne and Jerusalem artichokes. These figures were influenced by the presence of lucerne in the crop rotation, which has a high potential for N fixation compared to grass-clover. Thus, the N surplus was lowest in the *Free-range: grass-clover* scenario with 130 kg N ha^−1^. There were no substantial differences between the *Indoor finishing* scenario (143 kg N ha^−1^) and the *Free-range: alternative crops* scenario (139 kg N ha^−1^).

Regarding N losses, the alternative scenarios led to higher nitrous oxide emissions (6 kg N ha^−1^ compared to 3 kg N ha^−1^ in the *Indoor finishing* scenario), which were related to emissions taking place during grazing. In terms of ammonia emissions, the figures in the alternative scenarios were halved compared to the figures in the *Indoor finishing* scenario.

**Table 6:** Farm N balance (kg N ha^−1^) in three organic pig production systems.

	Indoor Finishing ^1^	Free-Range: Grass–clover ^2^	Free-Range: Alternative Crops ^3^
INPUT			
Imported feed	164	145	140
Seed	3	1	2
Straw	1	2	2
N fixation	31	38	51
N deposition	16	16	16
TOTAL INPUT	214	202	210
OUTPUT			
Live pigs	68	68	68
Culled sows	3	3	3
Dead animals	0	0	0
TOTAL OUTPUT	72	72	72
BALANCE	143	130	139
N losses			
Ammonia	49	24	20
Denitrification	3	6	6
Soil N	−8	4	4
N leaching	99	100	110
Indirect denitrification from leaching	1	1	1.1

^1^: Indoor finishing: Sows on pasture and growing-finishing pigs housed indoors; ^2^: Free-range: grass-clover: Sows on pasture and growing-finishing pigs foraging on grass-clover; ^3^: Free-range: alternative crops: Sows on pasture and growing-finishing pigs foraging on lucerne, grass-clover and Jerusalem artichokes.

Positive impacts could be observed in relation to soil N sequestration in the alternative scenarios due to the presence of grass–clover and lucerne fields. The highest values for N leaching were found in the *Free-range: alternative crops* scenario with 110 kg N ha^−1^, which represented an increase of 10% compared to the *Indoor finishing* scenario. The N leaching was similar for the *Indoor finishing system* and the *Free-range: grass-clover* system with 99 and 100 kg N ha^−1^. This was related to the fact that the *Free-range: grass-clover* system had a lower import of supplementary feed and a positive impact on soil N sequestration. The *Free-range: alternative crops* scenario had a worse performance compared to the *Free-range: grass-clover* system due to higher values for N fixation.

An important role in terms of N leaching was played by the various N losses from the systems. Previously, it was described that the N surplus was similar in the *Indoor finishing* and the *Free-range: alternative crops* scenarios. However, the different ammonia volatilization emission factors (Table 4) influenced the N leaching. Thus, in the *Indoor finishing* scenario, ammonia emissions were twice as high compared to those in the alternative scenarios and as a consequence the N leaching potential was reduced. Even though the emission factors from the alternative scenarios were similar, the N fixation in the *Free-range: grass-clover* scenario was lower compared to the *Free-range: alternative crops* scenario. This corroborated with a positive impact on soil N sequestration and therefore determined a lower level of N leaching in the *Free-range: grass-clover* scenario compared to the *Free-range: alternative crops* scenario, the former being similar to the *Indoor finishing* scenario.

**Table 7:** Greenhouse gas emissions in three organic pig production systems presented per kg live pig weight.

Contributor	Unit	Indoor Finishing ^1^	Free-Range: Grass–clover ^2^	Free-Range: Alternative Crops ^3^
I. Home-produced feed				
Nitrous oxide (N_2_O)	kg CO_2_ eq	0.46	0.84	0.75
Methane (CH_4_)				
from manure management	kg CO_2_ eq	0.41	0.05	0.04
Energy use (field operations)	kg CO_2_ eq	0.14	0.20	0.15
Total	kg CO_2_ eq	1.01	1.09	0.94
II. Imported feed				
From production of feed ^4^	kg CO_2_ eq	0.96	1.07	0.84
III. Enteric fermentation	kg CO_2_ eq	0.14	0.24	0.22
IV. Energy use	kg CO_2_ eq	0.06	0.00	0.00
Total (I+II+III+IV)	kg CO_2_ eq	2.17	2.4	2.00
V. Soil C emissions				
From imported feed	kg CO_2_ eq	0.21	0.21	0.16
From home-produced feed	kg CO_2_ eq	0.15	−0.08	−0.03
Total	kg CO_2_ eq	0.36	0.13	0.13
Land Use	m^2^ year	8.11	8.05	6.90
VI. Indirect Land Use Change	kg CO_2_ eq	1.16	1.15	0.99
TOTAL GHG emissions	kg CO_2_ eq	3.69	3.68	3.12

^1^: Indoor finishing: Sows on pasture and growing-finishing pigs housed indoors; ^2^: Free-range: grass-clover: Sows on pasture and growing-finishing pigs foraging on grass-clover; ^3^: Free-range: alternative crops: Sows on pasture and growing-finishing pigs foraging on lucerne, grass-clover and Jerusalem artichokes; ^4^: Refers to all categories of emissions related to feed production (nitrous oxide, methane and carbon dioxide emissions)

Halberg *et al.* (2010) [13] estimated N leaching ha^−1^ at farm level corresponding to 46 and 80 kg N ha^−^^1^ for an organic indoor finishing system similar to the indoor system in the present study and for an organic outdoor free-range system, respectively. These figures were much lower compared to the estimates in the present study. Even though the reported figures for N balances ha^−1^ were high in the study by Halberg *et al.* (2010) [13] with 125 and 164 kg N ha^−1^ for the indoor finishing and the free-range scenario, respectively, significant amounts of N were lost during denitrification (14–17 kg N ha^−1^ compared to 3–5 kg N ha^−1^ in the present study). The difference can be attributed to the method used for estimation [62], which refers to a higher emission factor for manure (1.25% of N amount in manure) compared to the updated IPCC presented in Table 4. In addition, soil N accumulation was 6–9 times higher in the scenarios modeled by Halberg *et al.* (2010) [13]. However, these figures refer to a 10 years horizon period, while in the present estimations the 100 years horizon was considered in agreement with the methodology described by Petersen *et al.* (2013) [53]. In this context, the differences between the N leaching could be explained by the different emission factors used in the present study and the study by Halberg *et al.* (2010) [13].

### 3.3. Greenhouse Gas Emissions per Kg Live Weight

Table 7 presents greenhouse gas (GHG) emissions (kg CO_2_ eq) per kg pig live weight (LW) and the main contributors: production of feed (nitrous oxide, methane and carbon dioxide emissions), enteric fermentation and energy use. The GHG emissions also refer to contributions from soil C changes and indirect land use change (iLUC), which are presented separately.

When soil C changes and iLUC were not included, the CF in the *Free-range: grass–clover* scenario was highest (2.40 kg CO_2_ eq kg^−1^ LW *versus* 2.17 and 2.00 kg CO_2_ eq kg^−1^ LW for the *Indoor finishing and the Free-range: alternative crops* scenarios, respectively). Feed production (on-farm produced and imported) was the largest contributor to GHG in all three scenarios, representing 89%–91% of total emissions. However, the contributing factors to the home produced feed were relatively different in the three scenarios. Whereas methane from manure handling was a significant contributor in the *Indoor finishing* scenario, this was not the case in the alternative scenarios. In the latter the contribution from nitrous oxide was higher, due to different ways of manure handling (collection and distribution *versus* deposited at the grazing area). Enteric fermentation and energy consumption during the production of organic pigs (e.g., MJ used in the indoor housing per slaughtered pig) only had minor contribution to CF kg^−1^ LW. Even though the number of produced pigs was similar in all three systems, the figures for enteric fermentation were higher in the alternative scenarios due to a higher forage intake in these systems compared to the *Indoor finishing* scenario.

Considering emissions related to soil C changes, differences existed between the *Indoor finishing* scenario and the two alternative scenarios (0.36 kg CO_2_ eq kg^−1^ LW for the *Indoor finishing* scenario compared to 0.13 kg CO_2_ eq kg^−1^ LW for the *Free-range: grass-clover* and the *Free-range: alternative crops* scenarios). They were given by the specific crop rotations used in the three systems. In the *Indoor finishing* scenario the emissions related to changes in soil carbon were due to imported and home produced feed, whereas in the alternative scenarios the impact related to imported feed was partly offset by the fact that carbon sequestration took place when producing on-farm feed including grass-clover or grass-clover and lucerne.

With regard to iLUC, the impact per produced kg LW pig was higher in the *Indoor finishing* scenario and the *Free-range: grass-clover* scenario (1.16 and 1.15 kg CO_2_ eq kg^−1^ LW, respectively) compared to the *Free-range: alternative crops* scenario with 0.99 kg CO_2_ eq kg^−1^ LW) due to the need for less import of feed in the latter. 

Taking into consideration soil C changes and iLUC the *Indoor finishing* and the *Free-range: grass-clover* scenarios had similar values of GHG emissions with 3.69 and 3.68 kg CO_2_ eq kg^−1^ LW, while GHG emissions kg^−1^ LW were considerably lower in the *Free-range: alternative crops* scenario (3.12 kg CO_2_ eq kg^−1^ LW). 

**Table 8:** Sensitivity analysis of three organic pig production systems presented in kg N ha^−1^ and kg CO_2_ eq. kg LW^−1^. The analysis was based on a 10% increase and decrease, respectively, of cereal yields. Reference refers to unchanged cereal yields.

Contributor	Unit	Indoor Finishing ^1^			Free-range: Grass–clover ^2^			Free-Range: Alternative Crops ^3^		
		Reference	−10%	+10%	Reference	−10%	+10%	Reference	−10%	+10%
**N leaching**	**kg N ha^−1^**	**99**	**105**	**94**	**100**	**102**	**91**	**110**	**112**	**107**
**Greenhouse gas emissions**										
**I. Home-produced feed**										
nitrous oxide (N_2_O)	Kg CO_2_ eq kg^−1^ LW	0.46	0.48	0.45	0.84	0.85	0.83	0.75	0.76	0.75
methane (CH_4_) from manure management	Kg CO_2_ eq kg^−1^ LW	0.41	0.40	0.43	0.05	0.05	0.04	0.04	0.04	0.04
energy use (field operations)	Kg CO_2_ eq kg^−1^ LW	0.14	0.13	0.15	0.20	0.19	0.21	0.15	0.15	0.16
Total	Kg CO_2_ eq kg^−1^ LW	1.01	1.01	1.03	1.09	1.09	1.08	0.94	0.95	0.95
**II. Imported feed**										
production of feed	Kg CO_2_ eq kg^−1^ LW	0.96	1.02	0.92	1.07	1.11	1.03	0.84	0.88	0.81
**III. Enteric fermentation**	**Kg CO_2_ eq kg^−1^ LW**	**0.14**	**0.15**	**0.14**	**0.24**	**0.18**	**0.21**	**0.22**	**0.22**	**0.22**
**IV. Energy use (stable)**	**Kg CO_2_ eq kg^−1^ LW**	**0.06**	**0.06**	**0.06**	**0.00**	**0.00**	**0.00**	**0.00**	**0.00**	**0.00**
**Total (I + II + III + IV)**	**Kg CO_2_ eq kg^−1^ LW**	**2.17**	**2.24**	**2.15**	**2.40**	**2.38**	**2.32**	**2**	**2.05**	**1.98**
**V. Soil C emissions**										
from imported feed	Kg CO_2_ eq kg^−1^ LW	0.21	0.22	0.19	0.21	0.22	0.20	0.16	0.15	0.15
from home-produced feed ^4^	Kg CO_2_ eq kg^−1^ LW	0.15	0.15	0.14	−0.08	−0.07	−0.07	−0.03	−0.03	−0.03
Total	**Kg CO_2_ eq kg^−1^ LW**	**0.36**	**0.37**	**0.33**	**0.13**	**0.15**	**0.13**	**0.13**	**0.12**	**0.12**
Land use	m^2^ year kg^−1^ LW	8.11	8.4	7.88	8.05	8.24	7.86	6.90	7.07	6.75
**VI. Indirect Land Use Change**	**Kg CO_2_ eq kg^−1^ LW**	**1.16**	**1.20**	**1.13**	**1.15**	**1.18**	**1.12**	**0.99**	**1.01**	**0.97**
**Total (I + II + III + IV + V + VI)**	**Kg CO_2_ eq kg^−1^ LW**	**3.69**	**3.81**	**3.61**	**3.68**	**3.71**	**3.57**	**3.12**	**3.18**	**3.07**

^1^: Indoor finishing: Sows on pasture and growing-finishing pigs housed indoors; ^2^: Free-range: grass-clover: Sows on pasture and growing-finishing pigs foraging on grass-clover; ^3^: Free-range: alternative crops: Sows on pasture and growing-finishing pigs foraging on lucerne, grass-clover and Jerusalem artichokes; ^4^: Refers to all categories of emissions related to feed production (nitrous oxide, methane and carbon dioxide emissions).

### 3.4. Sensitivity Analysis

Given the fact that the present analysis was based on modeling, several assumptions and estimates were made. The results are impacted by the overall crop yield as realized in the different scenarios where the balance between land use for cereal and other crops was varied. To illustrate the effect of change in crop yield a sensitivity analysis was conducted where the yields of cereals were increased or decreased by 10%, while yields of forage crops were kept unchanged. 

Table 8 shows the variation in N leaching (kg N ha^−1^) and GHG emissions (kg CO_2_ eq kg^−1^ LW) for the three organic pig production systems following assumed cereal yield.

A 10% decrease in cereal yield corresponded to a 2%–10% increase of the N leaching potential and GHG emissions in all scenarios and vice versa for a 10% increase in cereal yields. Regarding GHG emissions, the 10% decrease in cereal yields increased the contribution from imported feed, while an increase in yields led to slightly higher contributions from home-produced feed. The emissions due to enteric fermentation, energy use and soil C changes were relatively constant. Given the changes in the ratio of home-produced and imported feed, the impact on iLUC varied to a low extent (2%–3%).

Overall, the ranking of the systems did not change: The *Indoor finishing* and *Free-range: grass-clover* scenarios showed an improved performance in relation to N leaching and the *Free range: alternative crops* scenario had lower greenhouse gas emissions. However, an increase in cereals yield could represent an option to improve environmental performance of the *Free-range: grass-clover* system.

Another important assumption was the intake of forage by the pigs and the resultant production results. Among others, it was estimated that growing pigs were able to utilize forage crops corresponding to 3.7 MJ ME kg^−1^ weight gain (up to 18% on a DM basis) and finishers up to 8.5 MJ ME kg^−1^ weight gain (up to 22% on a DM basis) while assuming no significant impact on daily gain. We find that these assumptions were justified under the conditions of these production systems where an overall daily gain was around 800 g day^−1^ for growing-finishing pigs and where a restrictive feeding in any case is taking place in the last part of the fattening period to avoid excessive fat deposition and a reduced feed conversion rate. If, however, the reality would be a slightly lower daily gain of e.g., 5%–10% we assume this to have only minor influence on the results obtained. A matter of concern in this respect could be the land use required for grazing according to legislation. However, since the definition of an animal unit is based on the amount of nitrogen excreted in the manure, the way we modeled the systems, this is independent of growth rate. 

## 4. Conclusions

In terms of consumer expectations and animals being outdoors and able to perform species-specific behavior, the alternative scenarios had an advantage compared to the reference scenario. In addition, supposedly the alternative scenarios had agro-ecological advantages due to the improved crop rotation with grass-clover fields, lucerne and Jerusalem artichokes leading to improved nutrient recirculation, increased soil fertility, a higher diversity of crops and potentially a reduction of pest and diseases. However, regarding N leaching, all system showed high levels and only small differences were found, ranging from 99 to 110 kg N ha^−1^, the lowest figures being those of the *Indoor finishing* scenario. The *Free-range: alternative crops* scenario showed the poorest performance due to a high N input from biological fixation. Considering greenhouse gas emissions, the *Free-range: alternative crops* scenario was 8%–20% lower compared to the other two scenarios. When soil C emissions and iLUC were considered, the *Indoor finishing* and *Free-range: grass-clover* had similar impacts (3.69 and 3.68 kg CO_2_ eq kg^−1^ LW, respectively), while the impact of the *Free-range: alternative crops* system was 0.57 kg CO_2_ eq kg^−1^ LW lower. A sensitivity analysis based on a 10% decrease and increase, respectively in cereal yields did not change the overall ranking of the systems. We suggest that the alternative scenarios represent an actual possibility in terms of reducing the impact of organic pig production systems on climate change. However, in relation to N leaching various management options must be considered in order to improve system performance.

## Supplementary Files

Supplementary File 1
