# Supplementary material for: Increased Foraging in Outdoor Organic Pig Production—Modeling Environmental Consequences
Source: Foods. 2015 Nov 2;4(4):622–44. doi: 10.3390/foods4040622 (PMC5224558; doi:10.3390/foods4040622)
Supplement: Supplementary File 1 [file foods-04-00622-s001.docx]

Supplementary Material

| **Indoor finishing** |  | |  |  |  |
| --- | --- | --- | --- | --- | --- |
|  | **Pregnant,**  **dry sows, gilts** | | **Lactating sows** | **Weaners** | **Growing pigs 30-110 kg** |
| **Total feed, kg feedstuff per annual sow/per pig** |  | |  |  |  |
| **Composition of diet,** | **In percentage of total ration, kg feed** | | | | |
| Barley | 8.7 | | 26.0 | 37.3 | 41.8 |
| Oats | 36.4 | | 25.0 |  |  |
| Wheat |  | | 20.0 |  |  |
| Faba beans |  | | 8.0 | 14.8 | 13.9 |
| Peas |  | | 8.0 | 8.2 | 19.9 |
| Rape seed cake |  | | 8.0 | 25.0 | 12.2 |
| Rape seed oil |  | |  | 2.8 |  |
| Skimmed milk powder |  | |  | 10.0 |  |
| Soy bean cake |  | | 5.0 |  |  |
| Grass-clover | 33.0 | |  |  |  |
| Grass-clover silage | 21.9 | |  |  | 12.2 |
| **Total** | **100.0** | | **100.0** | **98.1** | **100.0** |
|  |  | |  |  |  |
|  | **Pregnant,**  **dry sows, gilts** | **Lactating sows** | | **Weaners** | **Growing pigs 30-110 kg** |
| **Total feed, kg DM per annual sow/per pig** | **800** | **947** | | **34** | **223** |
| **Composition of diet** | **Kg DM, %** | | | | |
| Barley | 18.0 | 25.7 | | 36.7 | 44.7 |
| Oats | 54.6 | 24.7 | |  |  |
| Wheat |  | 19.8 | |  |  |
| Faba beans |  | 8.2 | | 15.1 | 15.4 |
| Peas |  | 7.9 | | 8.1 | 21.3 |
| Rape seed cake |  | 8.3 | | 25.7 | 13.6 |
| Rape seed oil |  | 0.0 | | 3.2 |  |
| Skimmed milk powder |  | 0.0 | | 11.1 |  |
| Soy bean cake |  | 5.4 | |  |  |
| Grass-clover (MJ ME/kg DM: pigs = 10.5; sows = 12.2) | 14.8 |  | |  |  |
| Grass-clover silage (MJ ME/kg DM: pigs = 7.9; sows = 9.3) | 12.5 |  | |  | 5.0 |
| **Total** | **99.9** | **100.0** | | **100.0** | **100.0** |

|  | **Pregnant,**  **dry sows, gilts** | **Lactating sows** | **Weaners** | **Growing pigs 30-110 kg** |
| --- | --- | --- | --- | --- |
| **Total feed, MJ ME per annual sow/per pig** | **10,173** | **13,765** | **456** | **3094** |
| **Composition of diet** | **MJ ME, %** | | | |
| Barley | 22.0 | 27.1 | 35.3 | 47.1 |
| Oats | 56.4 | 22.1 |  |  |
| Wheat |  | 22.4 |  |  |
| Faba beans |  | 7.4 | 15.7 | 17.5 |
| Peas |  | 8.0 | 7.4 | 21.2 |
| Rape seed cake |  | 7.5 | 19.8 | 11.5 |
| Rape seed oil |  |  | 10.3 |  |
| Skimmed milk powder |  |  | 11.5 |  |
| Soy bean cake |  | 5.5 |  |  |
| Grass-clover (MJ ME/kg DM: pigs = 10.5; sows = 12.2) | 12.6 |  |  |  |
| Grass-clover silage (MJ ME/kg DM: pigs = 7.9; sows = 9.3) | 9.0 |  |  | 2.7 |
| **Total** | **100.0** | **100.0** | **100.0** | **100.0** |

| **Free-range: grass-clover** |  |  |  |  |  |
| --- | --- | --- | --- | --- | --- |
|  | **Pregnant,**  **dry sows, gilts** | **Lactating sows** | **Weaners** | **Growing pigs**  **30-50 kg** | **Growing pigs**  **50-110 kg** |
| **Total feed, kg feedstuff per annual sow/per pig** |  |  |  |  |  |
| **Composition of diet** | **In percentage of total ration, kg feed** | | | | |
| Barley | 28.1 | 26.0 | 47.0 | 23.8 | 28.0 |
| Oats |  | 25.0 |  | 23.1 | 7.3 |
| Wheat |  | 20.0 |  |  |  |
| Faba beans |  | 8.0 | 15.0 | 5.8 | 3.9 |
| Peas |  | 8.0 | 8.3 | 5.1 | 3.9 |
| Rape seed cake |  | 8.0 | 15.0 | 6.4 | 5.2 |
| Rape seed oil |  |  | 2.8 |  |  |
| Skimmed milk powder |  |  | 10.0 |  |  |
| Soy bean cake |  | 5.0 |  |  |  |
| Grass-clover | 28.4 |  |  | 7.2 | 10.3 |
| Grass-clover silage | 43.4 |  |  | 28.6 | 41.0 |
| **Total** | **99.9** | **100.0** | **98.1** | **100.0** | **99.6** |
|  |  |  |  |  |  |
|  | **Pregnant,**  **dry sows, gilts** | **Lactating sows** | **Weaners** | **Growing pigs**  **30-50 kg** | **Growing pigs**  **50-110 kg** |
| **Total feed, kg DM per annual sow/per pig** | **973** | **947** | **34** | **52** | **262** |
| **Composition of diet** | **kg DM, %** | | | | |
| Barley | 52.9 | 25.7 | 46.5 | 30.3 | 42.8 |
| Oats |  | 24.7 |  | 29.4 | 11.2 |
| Wheat |  | 19.8 |  |  |  |
| Faba beans |  | 8.2 | 15.4 | 7.7 | 6.1 |
| Peas |  | 7.9 | 8.2 | 6.5 | 7.7 |
| Rape seed cake |  | 8.3 | 15.5 | 8.5 | 10.5 |
| Rape seed oil |  |  | 3.2 |  |  |
| Skimmed milk powder |  |  | 11.2 |  |  |
| Soy bean cake |  | 5.4 |  |  |  |
| Grass-clover (MJ ME/kg DM: pigs = 10.5; sows = 12.2) | 15.9 |  |  | 3.5 | 4.1 |
| Grass-clover silage (MJ ME/kg DM: pigs = 7.9; sows = 9.3) | 31.2 |  |  | 14.0 | 17.5 |
| **Total** | **100.0** | **100.0** | **100.0** | **100.0** | **99.9** |
|  |  |  |  |  |  |
|  | **Pregnant,**  **dry sows, gilts** | **Lactating sows** | **Weaners** | **Growing pigs**  **30-50 kg** | **Growing pigs**  **50-110 kg** |
| **Total feed, MJ ME per annual sow/per pig** | **10,173** | **13,765** | **465** | **667** | **2807** |
| **Composition of diet** | **MJ ME, %** | | | | |
| Barley | 63.7 | 27.1 | 43.8 | 35.8 | 50.1 |
| Oats |  | 22.1 |  | 28.4 | 11.0 |
| Wheat |  | 22.4 |  |  |  |
| Faba beans |  | 7.4 | 15.6 | 9.8 | 6.1 |
| Peas |  | 8.0 | 7.3 | 7.3 | 6.6 |
| Rape seed cake |  | 7.5 | 11.7 | 8.1 | 7.8 |
| Rape seed oil |  |  | 10.2 |  |  |
| Skimmed milk powder |  |  | 11.3 |  |  |
| Soy bean cake |  | 5.5 |  |  |  |
| Grass-clover (MJ ME/kg DM: pigs = 10.5; sows = 12.2) | 14.2 |  |  | 2.2 | 3.8 |
| Grass-clover silage (MJ ME/kg DM: pigs = 7.9; sows = 9.3) | 22.1 |  |  | 8.6 | 14.6 |
| **Total** | **100.3** | **100.0** | **100.0** | **100.0** | **100.0** |

| **Free-range alternative crops** | |  |  |  |  |
| --- | --- | --- | --- | --- | --- |
|  | **Pregnant, dry sows, gilts** | **Lactating sows** | **Weaners** | **Growing pigs 30-50 kg** | **Growing pigs**  **50-110 kg** |
| **Total feed, kg feedstuff per annual sow/per pig** |  |  |  |  |  |
| **Composition of diet** | **In percentage of total ration, kg feed** | | | | |
| Barley | 13.6 | 26 | 47 | 60 | 40.5 |
| Oats |  | 25 |  |  | 10.7 |
| Wheat |  | 20 |  |  |  |
| Faba beans |  | 8 | 15 | 28.6 | 21.9 |
| Peas |  | 8 | 8.3 |  |  |
| Rape seed cake |  | 8 | 15 |  |  |
| Rape seed oil |  |  | 2.8 |  |  |
| Skimmed milk powder |  |  | 10 |  |  |
| Soy bean cake |  | 5 |  |  |  |
| Grass-clover | 21.6 |  |  |  | 2.7 |
| Grass-clover silage | 38.4 |  |  |  | 0.3 |
| Jerusalem artichokes | 26.4 |  |  | 4.8 | 13.6 |
| Lucerne |  |  |  |  | 2.8 |
| Lucerne silage |  |  |  | 6.7 | 7.5 |
| **Total** | **100** | **100** | **98.1** | **100.1** | **100** |
|  |  |  |  |  |  |
|  | **Pregnant and dry sows, gilts** | **Lactating sows** | **Weaners** | **Growing pigs 30-50 kg** | **Growing pigs 50-100 kg** |
| **Total feed, kg DM per annual sow/per pig** | **798** | **947** | **34** | **43** | **132** |
| **Composition of diet** | **kg DM, %** | | | | |
| Barley | 31.4 | 25.7 | 46.5 | 64.0 | 48.9 |
| Oats |  | 24.7 |  |  | 12.9 |
| Wheat |  | 19.8 |  |  |  |
| Faba beans |  | 8.2 | 15.4 | 31.6 | 27.5 |
| Peas |  | 7.9 | 8.2 |  |  |
| Rape seed cake |  | 8.3 | 15.5 |  |  |
| Rape seed oil |  |  | 3.2 |  |  |
| Skimmed milk powder |  |  | 11.2 |  |  |
| Soy bean cake |  | 5.4 |  |  |  |
| Grass-clover (MJ ME/kg DM: pigs = 10.5; sows = 12.2) | 19.1 |  |  |  | 1.0 |
| Grass-clover silage (MJ ME/kg DM: pigs = 7.9; sows = 9.3) | 34.0 |  |  |  | 0.1 |
| Jerusalem artichokes (MJ ME/kg DM: pigs = 14.6; sows = 15.4) | 15.4 |  |  | 1.3 | 4.1 |
| Lucerne (MJ ME/kg DM: pigs = 10.5; sows = 11.8) |  |  |  |  | 1.4 |
| Lucerne silage (MJ ME/kg DM: pigs = 7.9 ; sows = 9.3) |  |  |  | 3.1 | 4.0 |
| **Total** | **100.0** | **100.0** | **100.0** | **100.0** | **100.0** |
|  |  |  |  |  |  |
|  | **Pregnant,**  **dry sows, gilts** | **Lactating sows** | **Weaners** | **Growing pigs 30-50 kg** | **Growing pigs**  **50-100 kg** |
| **Total feed, ME per annual sow/per pig** | **10,173** | **13,765** | **465** | **667** | **2807** |
| **Composition of diet** | **MJ ME, %** | | | | |
| Barley | 40.5 | 26.1 | 44.0 | 63.4 | 54.0 |
| Oats |  | 21.3 |  |  | 11.7 |
| Wheat |  | 21.5 |  |  |  |
| Faba beans |  | 7.1 | 15.7 | 33.8 | 25.7 |
| Peas |  | 7.7 | 7.3 |  |  |
| Rape seed cake |  | 7.2 | 11.7 |  |  |
| Rape seed oil |  | 3.7 | 10.0 |  |  |
| Skimmed milk powder |  |  | 11.3 |  |  |
| Soy bean cake |  | 5.3 |  |  |  |
| Grass-clover (MJ ME/kg DM: pigs = 10.5; sows = 12.2) | 14.5 |  |  |  | 0.8 |
| Grass-clover silage (MJ ME/kg DM: pigs = 7.9; sows = 9.3) | 25.8 |  |  |  | 0.1 |
| Jerusalem artichokes (MJ ME/kg DM: pigs = 14.6; sows = 15.4) | 19.2 |  |  | 1.2 | 4.4 |
| Lucerne (MJ ME/kg DM: pigs = 10.5; sows = 11.8) |  |  |  |  | 1.1 |
| Lucerne silage (MJ ME/kg DM: pigs = 7.9 ; sows = 9.3) |  |  |  | 1.6 | 2.3 |
| **Total** | **100.0** | **100.0** | **100.0** | **100.0** | **100.1** |

© 2015 by the authors; licensee MDPI, Basel, Switzerland. This article is an open access article distributed under the terms and conditions of the Creative Commons Attribution license (http://creativecommons.org/licenses/by/4.0/).
